# Arterial collateral anatomy predicts the risk for intra-operative changes in somatosensory evoked potentials in patients undergoing carotid endarterectomy: a prospective cohort study

**DOI:** 10.1007/s00701-020-04624-y

**Published:** 2020-10-24

**Authors:** Mandy D. Müller, Kathleen Seidel, Giovanni Peschi, Eike Piechowiak, Pascal J. Mosimann, Philippe Schucht, Andreas Raabe, David Bervini

**Affiliations:** 1 Department of Neurosurgery, Inselspital, Bern University Hospital and University of Bern, 3010 Bern, Switzerland; 2 Department of Neuroradiology, Inselspital, Bern University Hospital and University of Bern, Bern, Switzerland

**Keywords:** Carotid endarterectomy, Neuromonitoring, Surgical morbidity, Cerebrovascular disease, Risks assessment, Vascular anatomy

## Abstract

**Background:**

During carotid endarterectomy (CEA), significant amplitude decrement of somatosensory evoked potentials (SEPs) is associated with post-operative neurological deficits.

**Objective:**

To investigate the association between an incomplete circle of Willis and/or contralateral ICA occlusion and subsequent changes in intra-operatively monitored SEPs.

**Methods:**

We performed a retrospective analysis of a single center, prospective cohort of consecutive patients undergoing CEA over a 42-month period after reviewing the collateral arterial anatomy on pre-operative radiological imaging. The primary endpoint was an intra-operative decline in SEPs > 50% compared to the baseline value during arterial cross-clamping. Univariate and multivariate logistic regression analyses were performed to investigate a potential association between contralateral ICA occlusion, incomplete circle of Willis, and subsequent alteration in SEPs.

**Results:**

A total of 140 consecutive patients were included, of which 116 patients (82.9%) had symptomatic carotid stenosis of at least 50% according to the classification used in the North American Carotid Surgery Trial (NASCET) (Stroke 22:711–720, 1991). Six patients (4.3%) showed contralateral ICA occlusion, 22 patients (16%) a missing/hypoplastic anterior communicating artery (Acom) or A1 segment, and 79 patients (56%) a missing ipsilateral posterior communicating artery (Pcom) or P1 segment. ICA occlusion and missing segments of the anterior circulation (missing A1 and/or missing Acom) were associated with the primary endpoint (*p* = 0.003 and *p* = 0.022, respectively).

**Conclusion:**

Contralateral ICA occlusion and missing anterior collaterals of the circle of Willis increase the risk of intra-operative SEP changes during CEA. Pre-operative assessment of collateral arterial anatomy might help identifying patients with an increased intra-operative risk.

## Introduction

Carotid stenosis is an important cause of stroke. Carotid endarterectomy (CEA) reduces the risk for ipsilateral stroke in patients with symptomatic and asymptomatic carotid stenosis. [8, 18] Generally, the stroke rate associated with CEA is low and has further decreased over the last years. [13] Cross-clamping is considered a critical phase during the procedure and may cause cerebral ischaemia, which is potentially preventable by insertion of a temporary shunt. [2] However, the insertion of a shunt to prevent cerebral ischaemia is not without risk and can cause thrombo-embolic events or dissection. [4] The risk for cerebral ischaemia during cross-clamping is determined by the collateral arterial network in the brain. [12] The circle of Willis is considered an essential collateral network of the entire brain. However, an incomplete circle of Willis is found in up to 50–80% of the general population. [12, 16, 22] Nevertheless, not all patients with an incomplete circle of Willis require a temporary shunt during cross-clamping in CEA. Furthermore, whether contralateral occlusion of the internal carotid artery (ICA) increases the risk of CEA is still controversial. [19, 20] In this retrospective analysis of a prospective cohort, we hypothesized that missing segments of the circle of Willis and/or contralateral ICA occlusion would significantly increase the risk of a decline in intra-operatively acquired somatosensory potentials (SEPs).

## Methods

This single center retrospective cohort study was approved by the local ethics committee (Protocol KEK-2019-00451, Bern). All patients with symptomatic or asymptomatic carotid stenosis admitted to our neurosurgical department between January 2012 and June 2015 were screened for inclusion. Patients presenting with symptoms consistent with a transitory ischaemic attack (TIA) including ocular symptoms or ischaemic stroke were considered symptomatic. All other patients were classified as asymptomatic. All patients undergoing CEA with intra-operative monitoring using median nerve somatosensory evoked potentials (SEPs) were included in the analysis. Patients with insufficient quality of pre-operative CTA or MRA were excluded (*N* = 4).

The primary endpoint of our analysis was a decline in intra-operatively acquired SEPs of > 50% for > 5 min after cross-clamping compared to the baseline value. [21]

### Somatosensory evoked potentials

The same method for intra-operative monitoring with SEPs was used as described previously. [21] In short, median nerve SEPs were performed by stimulation at the wrist with a pair of needle electrodes (Inomed Germany®). A single pulse stimulation with 0.5 ms pulse duration and a repetition rate ranging from 0.7 to 3.7 Hz was applied. Recording was performed via corkscrew electrodes placed according to the 10–20 EEG system on the patient’s scalp. For the right median nerve SEP C3´/Fz and for the left median nerve SEP C4´/Fz were chosen as standard derivation. Alternatively, Cz’ or the contralateral Cp’ served as reference to improve the signal to noise ratio and therefore the quality of recording. [10, 11] The responses were averaged 50 to 100 times. Amplitudes and latencies were measured and recorded in the protocol.

### Intra-operative recording of data

In each patient, median nerve SEPs were constantly monitored by a certified intra-operative monitoring (IOM) technician. The median nerve SEP amplitudes were recorded at least during:

- Baseline, before skin incision
- EEG burst suppression before cross-clamping of the internal carotid artery
- At time of internal carotid artery (ICA) cross-clamping
- 5 min after ICA clamping
- 10 min after ICA clamping
- Immediately after placement of a shunt (if placed)
- Re-opening (= reperfusion) of ICA

### Criteria for shunt placement

SEP amplitudes were measured at baseline and directly after cross-clamping. The pre-defined criterion for temporary shunting was > 5 min persisting > 50% reduction of SEP amplitude compared to the baseline value. In case of an amplitude change, SEPs were re-assessed 5 min after elevation of MAP to 120%, and directly after placement of the shunt. If a signal drop was already present at the time of EEG burst suppression and signals remained stable within a 15% range during ICA clamping, no temporary shunt was placed.

### Evaluation of circle of Willis and contralateral ICA

A standard stroke MR imaging protocol was performed, which included TOF-MRA and first-pass gadolinium-enhanced MRA of the cervical and intracranial arteries. The scans were acquired on 1.5T and 3T MR imaging systems (Magnetom Avanto and Magnetom Verio; Siemens, Erlangen, Germany). For the 1.5T scanner, the TOF parameters were the following: TR, 23 ms; TE, 7 ms; number of averages, 1; FOV read, 180 mm; FOV phase, 100%; voxel size, 0.9 × 0.7 × 1.2 mm; flip angle, 25°; acquisition time, 3 min 28 s. For the CE Carotid Angiography, the parameters were the following: TR, 2.59 ms; TE, 0.98 ms; FOV read, 360 mm; FOV phase, 75.0%; voxel size, 1.5 × 1.1 × 1.2 mm; flip angle, 29°; acquisition time, 1 min 21 s with 5–10 ml of Gadovist (gadobutrol, 1.0 mmol/mL, Bayer, Leverkusen, Germany – nonionic macrocyclic agent). For the 3T scanner, the TOF parameters were the following: TR, 22 ms; TE, 3.6 ms; number of averages, 1; FOV read, 180 mm; FOV phase, 100%; voxel size, 0.6 × 0.4 × 1.2 mm; flip angle, 20°; acquisition time, 3 min 45 s. For the CE Carotid Angiography, the parameters were the following: TR, 2.62 ms; TE, 0.95 ms; FOV read, 360 mm; FOV phase, 75.0%; voxel size, 1.3 × 1.1 × 1.4 mm; flip angle, 16°; acquisition time, 1 min 26 s. with 5–10 ml of Gadovist (gadobutrol, 1.0 mmol/mL, Bayer, Leverkusen, Germany – nonionic macrocyclic agent).

The CT-Angiography was acquired with a 128-section multidetector row CT scanner (Somatom Definition Edge; Siemens, Erlangen, Germany) with the patient in a head-first position, 1.5-s gantry rotation time, 0.6-mm collimation, and a pitch of 1.2, 512 × 512 matrix size. Automatic exposure control (CARE Dose 4D; Siemens Healthcare, Forchheim, Germany) automatically adjusted radiation output according to patient morphology. Forty milliliters of Iomeron400 (Bracco, Cadempino, Switzerland) was administered intravenously.

The supra-aortic arteries and configuration of the circle of Willis were assessed independently in each patient by two experienced neuroradiologists (GP, EIP) blinded to the intra-operative and clinical outcome of the patient. CTA or MRA was used for analysis depending on their availability. If both were available, CTA and MRA were compared and their respective quality assessed. In most cases, MRA was preferred due to superior quality of the image sets. Degree of ipsilateral and contralateral ICA stenosis was assessed according to the methodology used in the North American Carotid Surgery Trial (NASCET). [14] All components of the circle of Willis including the anterior communicating artery (Acom), the A1 segment of the anterior cerebral artery (A1) on both sides, the posterior communicating arteries on both sides (Pcom), and the P1 segment of the posterior cerebral arteries on both sides were assessed. A segment was considered hypoplastic if the diameter was at least 50% smaller compared to the contralateral side. If the segment was not visible on CTA and/or MRA, it was considered absent.

### Statistical analysis

Patients were divided in different groups according to the configuration of their circle of Willis: Group 1 contained all patients with either a missing or hypoplastic A1 segment (ipsi- or contralateral to the CEA) of the anterior cerebral artery or missing anterior communicating artery (Acom). Group 2 contained all patients with contralateral ICA occlusion. Group 3 contained all patients with either a missing (or hypoplastic) ipsilateral P1 segment of the posterior cerebral artery or missing (or hypoplastic) posterior communicating artery (Pcom) ipsilateral to the CEA. To investigate potential associations between the configuration of the circle of Willis and changes in intra-operatively monitored SEPs, univariate and multivariate logistic regression analyses were used. SPSS version 25.0 (IBM Corp, Chicago, IL) was used for all statistical analyses and an alpha level of 0.05 was defined to ascribe statistical significance.

## Results

In total, 144 consecutive patients who underwent CEA at our University Hospital between January 2012 and June 2015 were included in the analysis. All patients with pre-operative CTA and MRA who underwent CEA at our hospital and were monitored with SEPs intra-operatively were included in this analysis. Patients with insufficient quality of pre-operative CTA or MRA were excluded (*N* = 4). Of the remaining 140 patients included in this analysis, 116 patients (82.9%) had symptomatic carotid stenosis. Baseline characteristics of patients included in our analysis are detailed in Table 1.

**Table 1:** Baseline characteristics stratified by configuration of the circle of Willis

	Group 1 (missing/hypoplastic Acom or A1) *n* = 22 (16%)	Group 2 (contralateral ICA occlusion) *n* = 6 (4%)	Group 3 (missing ipsilateral Pcom or P1) *n* = 79 (56%)	Overall *n* = 140 (100%)
Age (mean)	72	65	71	71
Gender				
Male	18 (82%)	6 (100%)	65 (81%)	104 (74%)
Female	4 (18%)	0	14 (19%)	36 (26%)
Left sided stenosis	11 (50%)	3 (50%)	43 (54%)	71 (51%)
Symptomatic carotid stenosis	17 (77%)	4 (67%)	70 (87%)	116 (83%)
Degree of ipsilateral carotidstenosis				
- < 50%	- 0	- 0	- 2	- 2
- 50–70%	- 9	- 4	- 28	- 54
- > 70%	- 13	- 2	- 49	- 84

Baseline characteristics of patients included in our analysis according to the configuration of their circle of Willis and contralateral ICA

**Fig. 1:**
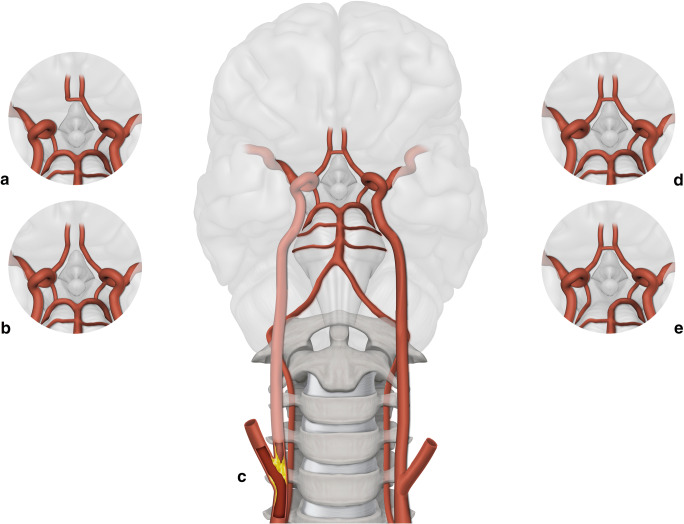
Arterial collateral anatomy. Configuration of the circle of Willis and contralateral internal carotid artery. (a) and (b) show missing anterior collaterals in the circle of Willis (a: missing A1 segment of the anterior cerebral artery, b: missing anterior communicating artery). (c) shows contralateral carotid occlusion. (d) and (e) show missing posterior collaterals (d: missing posterior communicating artery, e: missing P1 segment of the posterior cerebral artery)

Overall, 95 patients (65%) showed an incomplete circle of Willis on pre-operative CTA or MRA, and six patients (4.3%) showed contralateral ICA occlusion. Examples of the different configurations of the circle of Willis in our study population are shown in Fig. 1. Overall, ten patients (7.1%) showed a SEP amplitude decrement of > 50%. Seven of these patients displayed a persisting SEP amplitude decline for > 5 min after cross-clamping (primary endpoint). Three patients already experienced an SEP amplitude decrement at the time of EEG burst suppression and remained stable within a 15% range during carotid cross-clamping.

In our study population, six (4.3%) patients received a temporary shunt. Five patients received a shunt due to SEP amplitude decrement and one patient due to loss of flow signal in the ipsilateral middle cerebral artery monitored by transcranial Doppler ultrasound.

Contralateral ICA occlusion was significantly associated with a decline in intra-operative SEP s > 0.5 (OR 12.9, 95% CI 1.90–87.83, *p* = 0.009). Assessing the configuration of the circle of Willis, missing segments of the anterior circulation (missing ipsilateral or contralateral A1 segment and/or missing Acom) showed a trend towards a significantly increased risk of a decline in intra-operative SEPs >0.5 without reaching the pre-defined level of statistical significance (OR 4.5, 95% CI 0.93–21.71, *p* = 0.061). However, missing segments in the posterior circulation (ipsilateral posterior communicating artery or P1 segment of the posterior cerebral artery; OR 4.9, 95% CI 0.58–42.10, *p* = 0.145) were not associated with the primary endpoint.

Our multivariate model including contralateral ICA occlusion and missing segments of the anterior circulation (missing A1 segment and/or missing Acom) confirmed our previous results showing a significant association between contralateral ICA occlusion and intra-operative SEP changes during cross-clamping, while the association between missing segments of the anterior circulation and the primary outcome also reached statistical significance (*p* = 0.003, and *p* = 0.022 respectively; Table 2).

**Table 2:** univariate and multivariate model

	*n*/*N*	Univariate model			Multivariate model		
		OR	95% confidence interval	*p* value	OR	95% confidence interval	*p* value
Group 1 (missing Acom and/or A1)	3/22	4.5	0.93–21.71	0.061	8.7	1.36–55.47	0.022
Group 2 (contralateral ICA occlusion)	2/6	16.1	2.25–115.34	0.006	27.5	3.01–248.0	0.003
Group 3 (missing ipsilateral Pcom and/or P1)	6/79	4.9	0.58–42.01	0.145	–	–	–

Univariate and multivariate regression analysis depicting the association between the configuration of the circle of Willis and decline in somatosensory evoked potentials (SEPs). *Acom*, anterior communicating artery; *A1*, A1 segment of the anterior cerebral artery; *ICA*, internal carotid artery; *n*, number of patients with significant SEP decline in respective group; *N*, number of patients in respective group; *OR*, odds ratio; *Pcom*, posterior communicating artery; *P1*, P1 segment of the posterior cerebral artery

## Discussion

In this retrospective analysis, we were able to show a significant association between contralateral ICA occlusion, missing segments of the anterior circulation, and a decline in intra-operative SEPs > 50% amplitude value compared to the baseline value.

Nowadays, CEA can be performed under general or under local anesthesia. However, surgery under local anesthesia carries its own set of risks (e.g., failure of local anesthesia or respiratory complications due to diaphragmatic or vocal cord paralysis) and urgent conversion to general anesthesia may be necessary. [7] Therefore, many surgeons opt for performing CEA in general anesthesia, which showed similar results compared to CEA performed in local anesthesia. [6] When performing CEA under general anesthesia, monitoring of the cerebral function is mandatory to determine which patients will need a shunt due to insufficient cerebral perfusion during cross-clamping and which patients have sufficient collaterals and can safely be operated without shunting. Today, different monitoring methods exist. [1] The use of SEPs has been shown to be safe and associated with very low post-operative stroke rates. [15] Our center routinely uses SEP monitoring and selective shunting while performing CEA. In a previous study, we were able to show that SEP monitoring is superior to the use of transcranial Doppler to predict post-operative neurological deficits. [21]

Cross-clamping during the procedure is considered a critical phase during which the risk for cerebral ischemia is particularly high. However, predictors for the necessity of inserting a temporary shunt during this phase of the procedure are currently not well established and the insertion of a shunt carries its own risks such as dissection or thrombo-embolic events. [4] Compared to previous studies, the shunt rate in our study population was comparatively low. [16, 17] Nevertheless, as published in a previous report, the stroke or death rate at our center stayed considerably below the recommended stroke or death rates for carotid endarterectomy. [21] The circle of Willis is an important collateral network of the brain and missing segments may increase the risk for decreased perfusion and therefore increase the need for a temporary shunt to avoid cerebral ischemia during cross-clamping. Our study confirms this notion as missing segments of the circle of Willis and contralateral ICA occlusion were significantly associated with intra-operative SEP changes. These results are supported by a previous study investigating the configuration of the circle of Willis and the intolerance of cross-clamping during CEA. [12] Nevertheless, an incomplete circle of Willis is common and occurred in > 60% in our study population. This corresponds with the percentage described in the literature. [3] Most of these patients did not show a significant decrease of intra-operative SEPs and did not require a temporary shunt. Whether contralateral ICA occlusion increases the risk of CEA is still a matter of debate. [5, 19] Our study showed a significant association between contralateral ICA occlusion and intra-operative SEP decrement suggesting that these patients are at higher risk to require a temporary shunt during cross-clamping.

A previous study showed that in patients with severe carotid stenosis, the anterior collateral network seems to be more important to maintain cerebral perfusion than the posterior circulation. [9] The results of our analysis concur with these findings as only missing segments of the anterior circulation showed an association with a decline in intra-operative SEPs. Hence, pre-operative assessment of the circle of Willis and contralateral carotid artery might help identify patients at increased risk of intra-operative SEP decline and candidates for the insertion of a temporary shunt during cross-clamping.

### Limitations

Our study has some limitations. First, despite the prospective nature of the cohort, the analysis was performed retrospectively and the sample size was limited. However, to the best of our knowledge, this is the first study investigating the association between contralateral ICA occlusion, missing segments of the circle of Willis, and intra-operative SEP changes during CEA. Further prospective research is needed to confirm the association between contralateral ICA occlusion, missing segments of the circle of Willis, and intra-operative SEP changes. Moreover, studies investigating the configuration of the circle of Willis and its potential influence not only on SEP changes but also on clinical outcome after CEA are needed. Our study was not powered to answer these questions.

In addition, because of the sample size and limited number of relevant SEP changes in our study population, the power of our analyses was comparatively low. Nevertheless, our results were confirmed in a multivariate model including all three patient groups.

## Conclusion

We were able to show that contralateral ICA occlusion and missing segments in the anterior circulation, but not the posterior circulation of the circle of Willis, increase the risk of intra-operative SEP changes during carotid endarterectomy. Pre-operative assessment of the contralateral carotid artery and the circle of Willis might therefore help predicting the need for an intra-operative carotid shunt and identifying patients with an increased intra-operative risk.
